# Deep Learning for Dynamic Prognostic Prediction in Minimally Invasive Surgery for Intracerebral Hemorrhage: Model Development and Validation Study

**DOI:** 10.2196/86327

**Published:** 2026-01-07

**Authors:** Jingxuan Wang, Jian Shi, Qing Ye, Danyang Chen, Yuhao Sun, Chao Pan, Yingxin Tang, Ping Zhang, Zhouping Tang

**Affiliations:** 1 Department of Neurology Tongji Hospital, Tongji Medical College Huazhong University of Science and Technology Wuhan City China; 2 State Key Laboratory of Intelligent Manufacturing Equipment and Technology Huazhong University of Science and Technology Wuhan City China; 3 Big Data and Artificial Intelligence Office Tongji Hospital, Tongji Medical College Huazhong University of Science and Technology Wuhan City China; 4 Key Laboratory for Diagnosis and Treatment of Severe Zoonotic Infectious Diseases Huazhong University of Science and Technology Wuhan city China

**Keywords:** intracerebral hemorrhage, minimally invasive surgery, dynamic features, prognosis prediction, deep learning

## Abstract

**Background:**

The pathological and physiological state of patients with intracerebral hemorrhage (ICH) after minimally invasive surgery (MIS) is a dynamic evolution, and the traditional models cannot dynamically predict prognosis. Clinical data at multiple time points often show the characteristics of different categories, different numbers, and missing data. The existing models lack methods to deal with imbalanced data.

**Objective:**

This study aims to develop and validate a dynamic prognostic model using multi–time point data from patients with ICH undergoing MIS to predict survival and functional outcomes.

**Methods:**

In this study, 287 patients who underwent MIS for ICH were retrospectively collected on the day of surgery, days 1, 3, 7, and 14 after surgery, and the day of drainage tube removal. Their general information, vital signs, laboratory test findings, neurological function scores, head hematoma volume, and MIS-related indicators were collected. In addition, this study proposes a multistep attention model, namely the MultiStep Transformer. The model can simultaneously output 3 types of prediction probabilities for 30-day survival probability, 180-day survival probability, and 180-day favorable functional outcome (modified Rankin Scale [mRS] 0-3) probability. Five-fold cross-validation was used to evaluate the performance of the model and compare it with mainstream models and traditional scores. The main evaluation indexes included accuracy, precision, recall, and *F*_1_-score. The predictive performance of the model was evaluated using receiver operating characteristic (ROC) curves; its calibration was assessed via calibration curves; and its clinical utility was examined using decision curve analysis (DCA). Attributable value analysis was conducted to assess the key predictive features.

**Results:**

The 30‑day survival rate, 180‑day survival rate, and 180‑day favorable functional outcome rate among 287 patients were 92.3%, 88.8%, and 52.3%, respectively. In terms of predictive efficacy for survival and functional outcomes, the MultiStep Transformer model showed a remarkable superiority over traditional scoring systems and other deep learning models. For these three outcomes, the model achieved areas under the receiver operating characteristic curves (AUROCs) of 0.87 (95% CI 0.82-0.92), 0.85 (95% CI 0.77-0.93), and 0.75 (95% CI 0.72-0.78), with corresponding Brier scores of 0.1041, 0.1115, and 0.231. DCA confirmed that the model provided a definite clinical net benefit when threshold probabilities ranged within 0.06-0.26, 0.04-0.5, and 0.21-0.71.

**Conclusions:**

The MultiStep Transformer model proposed in this study can effectively use imbalanced data to construct a model. It possesses good dynamic prediction ability for short-term and long-term survival and functional outcome of patients with ICH undergoing MIS, providing a novel tool for individualized assessment of prognosis among patients with ICH undergoing MIS.

## Introduction

Intracerebral hemorrhage (ICH) accounts for 10%-15% of all stroke cases [1,2]. It is the leading cause of death and disability worldwide [3], characterized by [4] high morbidity, disability, and mortality rates. Conservative medical treatment of ICH exhibits limited efficacy [5]. Surgery for the removal of hematoma may improve the functional outcome of patients [6]. However, Surgical Trial in Intracerebral Haemorrhage (STICH) [7] and Surgical Trial in Intracerebral Haemorrhage II (STICH II) [8] confirmed the failure of craniotomy in improving functional outcomes among patients with ICH.

Recently, minimally invasive surgery (MIS), characterized by limited trauma, rapid recovery, and other advantages, has received increasing attention. Minimally Invasive Surgery Plus Alteplase for Intracerebral Hemorrhage Evacuation Phase 3 (MISTIE Ⅲ) [9] demonstrated the safety of minimally invasive hematoma evacuation combined with alteplase thrombolysis. The Early Minimally Invasive Removal of Intracerebral Hemorrhage (ENRICH) [10] trial reported that MIS can improve the functional outcomes of patients with lobar ICH at 180 days. Although the ENRICH trial provided encouraging evidence regarding the efficacy of MIS, not all patients with ICH can benefit from this approach. The uncertainty surrounding the therapeutic efficacy of MIS poses challenges for assessing the prognosis of patients.

Moreover, the condition of patients with ICH is a dynamic process. Although some tools have been used to predict death or functional outcome in patients with ICH [11-17], these predictions are mostly based on data at admission or a single time point, which cannot reflect the dynamic changes of the disease and also limit the accuracy of prognostic assessment. Electronic health records (EHRs) containing abundant data reflect the pathological and physiological state of dynamic changes in patients with ICH undergoing MIS. However, data extracted from EHR, without systematic organization, exhibits characteristics such as variable quality, high dimensionality, heterogeneity, temporality, and incompleteness [18], posing significant challenges for predictive modeling. Therefore, this study aimed to leverage multitime point, imbalanced data from patients undergoing MIS for ICH to develop a dynamic prognostic model and validate its performance in predicting survival and functional outcomes.

## Methods

### Study Design and Population

Patients with ICH who underwent MIS for ICH in the Department of Neurology, Tongji Hospital, Tongji Medical College, Huazhong University of Science and Technology were enrolled in this study. Clinical and imaging data were collected retrospectively on the day of surgery, 1, 3, 7, and 14 days after surgery, and on the day of drainage tube removal. A deep learning algorithm was used to construct a dynamic prognostic model and validate the effect of the model.

**Figure 1:**
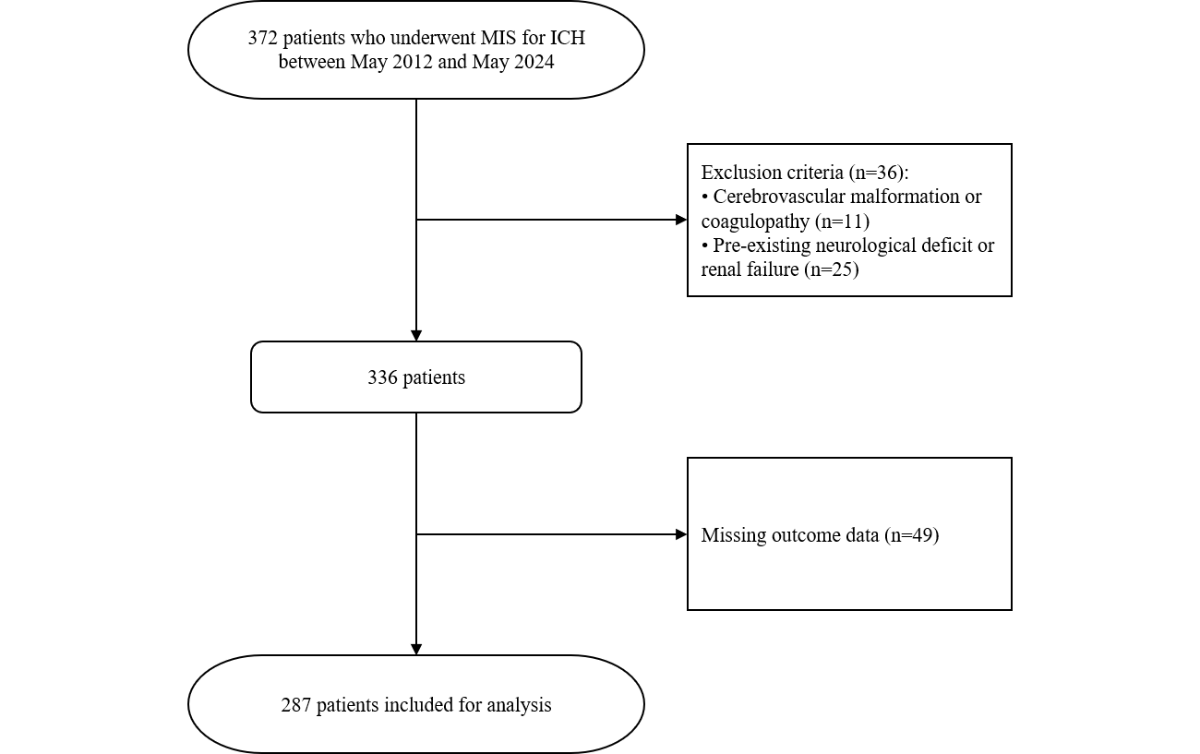
Patient selection flowchart. ICH: intracerebral hemorrhage; MIS: minimally invasive surgery.

In total, 372 patients undergoing MIS for ICH between May 2012 and May 2024 were selected. Among them, 11 cases of ICH caused by cerebrovascular malformation and coagulopathy, 25 cases with a history of neurological deficit and renal failure, and 49 patients with missing outcome data were excluded, and data from 287 patients were analyzed. A flowchart for the patient selection process is shown in Figure 1.

The inclusion criteria were as follows: (1) patients with spontaneous acute ICH in basal ganglia or lobar regions based on cranial computed tomography (CT), who underwent stereotactic intracranial hematoma puncture and drainage surgery after admission; (2) ICH hematoma volume 20-100 mL; (3) age 18-80 years old; (4) surgery within 1 week of onset; and (5) survival for at least 7 days after admission.

The exclusion criteria were as follows: (1) coagulation disorders, such as thrombocytopenia and significant abnormal coagulation indexes caused by hepatitis; (2) traumatic ICH; (3) intracranial infection and other diseases leading to a significant increase in intracranial pressure; (4) previous severe heart, liver, kidney, or lung diseases or functional failure; (5) previous history of neurological dysfunction; (6) ICH was caused by structural abnormalities of the cerebral blood vessels (eg, arteriovenous malformations, intracranial aneurysms, vasculitis, and moyamoya disease); and (7) ICH was caused by brain tumor or cerebral infarction and was treated with thrombolytic therapy.

The model was developed and validated in accordance with the TRIPOD-AI (Transparent Reporting of a multivariable prediction model for Individual Prognosis Or Diagnosis–Artificial Intelligence) checklist.

### Data Sources and Collection

We electronically extracted clinical data from the EHR of Tongji Hospital, Tongji Medical College, Huazhong University of Science and Technology. Data were collected on general information, vital signs, laboratory test findings, neurological function scores, and MIS-related metrics, such as the time from onset to surgery and the duration of drainage tube placement, along with established neurological scales: the ICH score, Glasgow Coma Scale (GCS), National Institutes of Health Stroke Scale (NIHSS), and the functional outcome in patients with primary intracerebral hemorrhage (FUNC) score. Rapid segmentation and volume calculation of hematomas on cranial CT images were conducted using 3D Slicer.

### Follow-up and Clinical Outcomes

Follow-up was conducted via telephone call. The primary outcome was all-cause mortality at 30 days. Secondary outcomes included all-cause mortality at 180 days and functional outcome at 180 days. The outcomes were assessed using the modified Rankin Scale (mRS), which was transformed into a binary variable, favorable functional outcome (mRS 0-3) and unfavorable functional outcome (mRS 4-6) [19,20]. The assessment was conducted by 2 medical doctors (JW and YS), with discrepancies being resolved through arbitration by a senior physician (PZ).

### Data Processing and Statistical Analysis

Missing values in this study were predominantly attributed to the absence of imaging features at certain time points, whereas clinical features (vital signs, laboratory studies, etc) were fully documented. Instead of imputing the missing imaging features, the model automatically ignored the corresponding time points via an attention mechanism and completed representation learning and outcome prediction solely based on available temporal imaging features and comprehensive clinical features. To ensure a consistent input structure across patients and time points, we adopted a simple zero-imputation strategy during preprocessing. For patients with missing measurements at specific time points (eg, when certain laboratory tests or clinical assessments were not performed on a given day), the corresponding feature values were set to zero. All features were standardized before model training so that zero-imputed values lie within a well-defined range and are distinguishable from typical observed values.

For continuous variables, intergroup comparisons were conducted using the *t* test. The chi-square test was used for categorical variables. Data are presented as median (IQR), mean (SD), and range. A *P* value of <.05 was considered statistically significant.

### Model Development and Training

#### Modeling Strategy

The changes of clinical indicators after MIS for ICH reflect the dynamic changes of individual status. Reasonable modeling of time series data dependence and cross-time point information interaction is of high importance for survival and prognosis prediction. We collected a data series of patients’ multidimensional clinical measures: 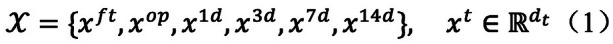 , where *x*^1^*^d^*, *x*^3^*^d^*, *x*^7^*^d^*, *x*^14^*^d^* denote the characteristics at 1, 3, 7, and 14 days after surgery, respectively. *x^ft^* denotes fixed features (eg, gender and age) that do not change over time. *x^op^*denotes the characteristics of the day of surgery. In particular, since not all individuals had the same drainage tube removal time, the corresponding characteristics were introduced separately.

Our objective was to learn a mapping function *f* : *X* → *γ*, where γϵ{0,1}represents the survival and prognosis labels, specifically defined as 30-day survival, 180-day survival, and 180-day favorable functional outcome. Crucially, we aimed to establish a mapping function capable of predicting both future data and the final labels from postoperative data of any given day. For instance, when input with first-day postoperative data *x*^1^*^d^*, the model was meant to successfully predict subsequent data *x*^3^*^d^*, *x*^7^*^d^*, *x*^14^*^d^* and the final ternary label γ. The problem was a multistage, multi–time point, and multivariate time series classification task.

Since the clinical indicators at each time point in *X* were not entirely consistent, it was not feasible to establish a unified model for cyclic prediction at each time point. Available machine learning or deep learning models exhibit limited capability in handling variable dimensional feature data [21]. To address this, we proposed a MultiStep Transformer model designed to capture nonlinearly evolving clinical features over time and manage the cross-influences existing between different time points.

#### MultiStep Transformer Model Architecture

**Figure 2:**
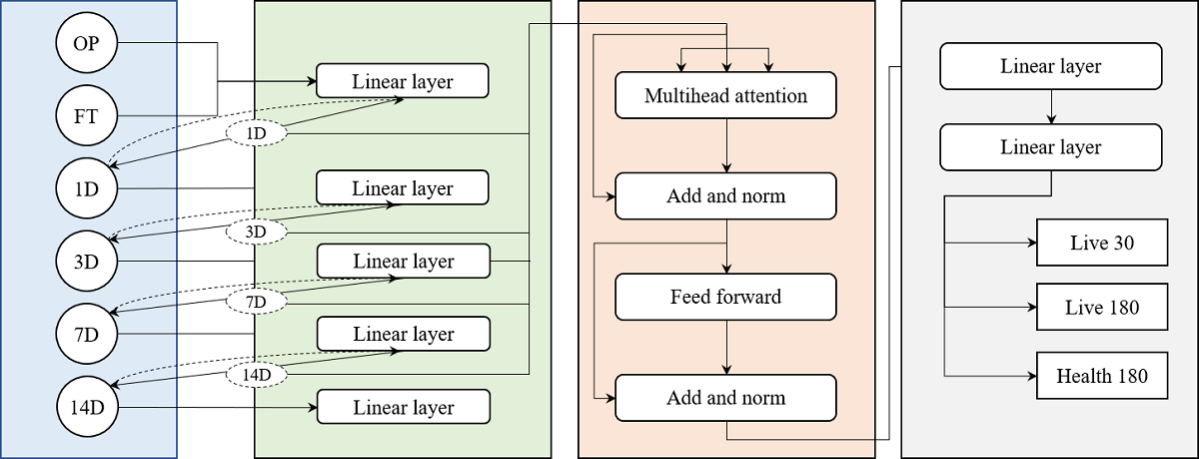
Model diagram.

In the MultiStep Transformer model, we introduced a multistage feature prediction module and a time-sharing embedding mechanism. It cooperated with the transformer encoder to realize the reinforcement modeling of multi–time point sequences. Specifically, we constructed a multilayer feature prediction module to predict and train each prognostic data separately. Finally, the Transformer encoder was used to predict 30-day survival probability, 180-day survival probability, and 180-day favorable prognosis probability. The overall process of the model is shown in Figure 2.

The MultiStep Transformer model was able to receive data at any time point as input and output for 3 types of prediction probabilities. The model framework is as follows:

#### Multistage Feature Cycle Prediction Module

The core idea of the multistage feature cycle prediction module was to use the information of current and previous time points to recursively infer the feature expression of missing or future time points, which was formulated as



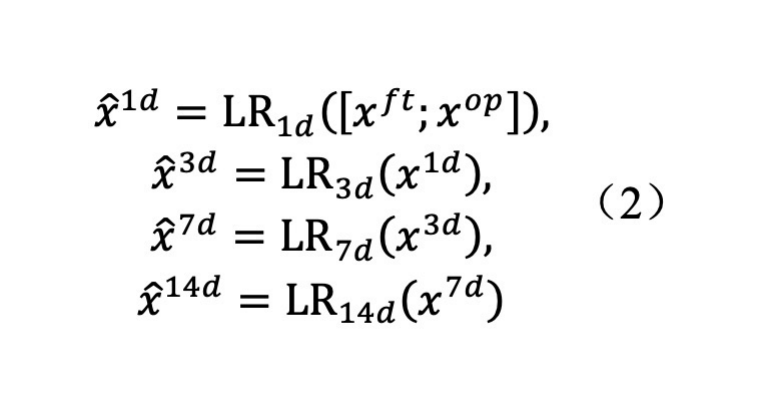



Here, LR*_t_* (∙)represents a linear mapping layer specific to the t-th time point, responsible for establishing recursive dependencies among the time-series features. This design effectively mitigated the challenges of incomplete clinical data and the isolation of temporal features, thereby enhancing the ability of the model to capture temporal evolutionary trends.

#### Time-Sharing Feature Embedding

In view of the large difference of input data dimensions and strong heterogeneity of feature distribution at different time points, an independent linear mapping layer was designed:



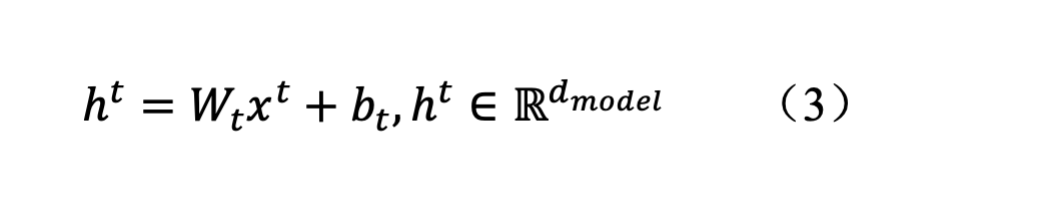



The high-dimensional projection of the input at each time point was conducted to eliminate the difference in input dimensions and ensure the consistency of the sequence dimensions of the input of the transformer encoder. In addition, the independent mapping parameters *W_t_* and *d_t_* captured the unique statistical characteristics of time points and improved the adaptability of the model to the information of different stages.

#### Transformer Encoder Structure and Time-Series Dependence Modeling

Through the self-attention mechanism, the transformer encoder flexibly modeled the interactive dependencies between all positions in a sequence. It overcame the limitations of traditional recurrent neural networks and possessed the capabilities of parallel computation and long-range dependency capture. In our framework, the same temporal feature sequence was used simultaneously as the query, key, and value in the self-attention operation. This design allows feature extraction to be performed directly on the observed sequence without forcing all patients to have the same number of time points and therefore naturally supports variable-length temporal inputs.

Input sequence: concatenate the embedding vectors at all time points to form an input sequence:



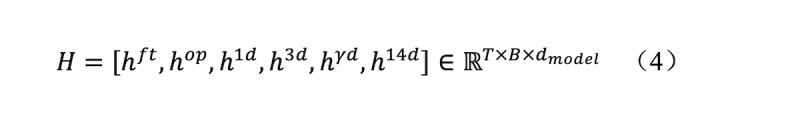



Here, *t*= 6 represents the number of input feature layers, and B denotes the batch size for each training or inference step. The experimental framework supports arbitrary combinations of time points as input, for example using only Day‑1 data or Days 1, 3, 7, and 14, depending on data availability and clinical requirements.

Encoding process: the multilayer stacked transformer encoder consisted of multihead self-attention and a feed-forward network. A single layer was formulated as



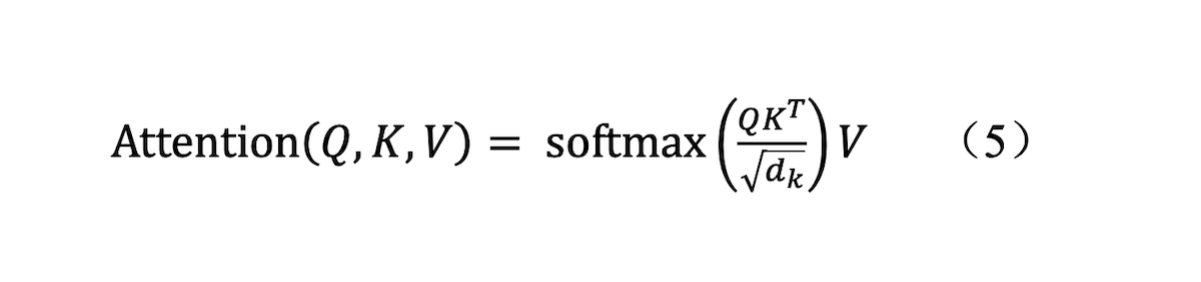



Where the same sequence serves as Q, K, and V in the self-attention module. The model performs multistep, progressive computation; features from earlier time points are encoded first, and then information from subsequent time points is gradually integrated. After multilayer coding, the model comprehensively considered the influence between different time points and its feature correlation, and the output sequence was expressed as:

Z = TransformerEncoder(*H*) (6)

The encoding vector *Z^d^* at the final time point of the sequence was taken as the representation of the comprehensive temporal dynamic state of patients.

#### Survival Prediction Head

The final classification layer was mapped using single-layer linearity:



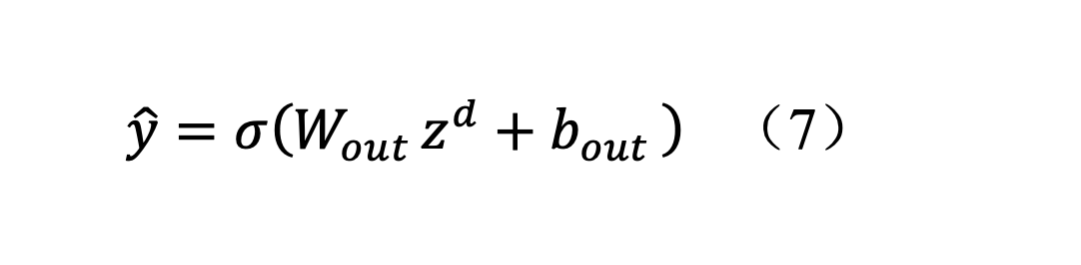



Here, σ denotes the sigmoid activation function for generating binary classification probability outputs. The output data format was (30 live, 180 live, and 180 heal), representing the probability of 30-day survival, 180-day survival, and 180-day favorable functional outcome, respectively.

#### Training and Prediction Processes

For model training, data were input uniformly, and the model was trained step by step. Specifically, features from all time points *X* = {*x^ft^*, *x^op^*, *x*^1^*^d^*, *x*^3^*^d^*, *x*^7^*^d^*, *x*^14^*^d^*}were input into the model. The model first processed the features [*x^ft^*, *x^op^*] through the linear layer LR_1_*_d_* to compute 
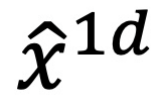
, and calculate the loss using *x*^1^*^d^*, followed by backpropagation to optimize LR_1_*_d_*. Subsequently, stepwise prediction and optimization were conducted sequentially based on the mapping layers in the multistage feature prediction module. All features, including known inputs and predicted outputs, were combined into a complete feature sequence 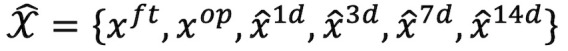. Finally, the transformer encoded all temporal features to obtain the patient’s dynamic state representation Z, which was then classified using a linear layer.

During inference, given the first *i* temporal features *X_i_* (eg, *X_i_* = {*x^ft^*, *x^op^*, *x*^1^*^d^*, *x*^3^*^d^*}), the model applied masking to the existing feature time points based on the number of feature layers and directly computed subsequent features 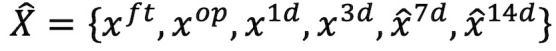, finally predicting survival and prognosis (30 live, 180 live, and 180 heal).

Model hyperparameters were selected empirically based on preliminary experiments on the training folds. The network was optimized using the Adam optimizer with a fixed learning rate of 0.001 and trained for 200 epochs per fold. We used the cross-entropy loss for outcome prediction, combined with the feature prediction loss for intermediate temporal features in the multistage prediction module. The batch size, number of transformer layers, hidden dimensions, and dropout rate were tuned within a reasonable range to balance model performance and overfitting risk.

### Model Evaluation

The predictive performances of different models and scores were compared using accuracy, precision, recall, and *F*_1_-score. The predictive performance of the model was evaluated by the area under the receiver operating characteristic curve (AUROC). Calibration curves were plotted to assess the consistency between the predicted probabilities and the observed outcomes of the model. In addition, decision curve analysis (DCA) was performed to evaluate the clinical utility of the model.

### Model Interpretation

To gain insight into the decision-making process of the model, we used attribution values, a technique used to quantify how much each input feature contributes to the output of the model. Attribution values revealed the importance of clinical characteristics in predicting the outcome and helped us determine which characteristics had the greatest impact on the model’s decision-making process.

### Ethical Considerations

This study was approved by the Medical Ethics Committee of Tongji Hospital, Tongji Medical College, Huazhong University of Science and Technology (TJ-IRB202502129). Given the retrospective nature of the study and the absence of any direct interaction with participants, informed consent was exempted by the ethics committee, and no compensation of any form was provided. This study adheres to the ethical principles outlined in the Declaration of Helsinki.

## Results

### Baseline Characteristics of the Study Population

The mean age of the 287 patients was 53.2 (10.6) years, and 67.9% of the patients were male. Their 30-day survival rate was 92.3%, their 180-day survival rate was 87.8%, and the rate of favorable functional outcome at 180 days was 52.3%. The presence of intraventricular hemorrhage, GCS, NIHSS, ICH score, and FUNC score showed significant differences across the 3 groups of patients (Tables S1-S3 in Multimedia Appendices 1-3).

### Evaluation of the Model Performance

Due to variations in clinical assessment items across different time points, the feature categories at each time point were not entirely consistent. In the collected dataset, there were 48 features on the day of surgery, 47 features on postoperative days 1 and 3, a total of 46 features on postoperative day 7, a total of 10 features on postoperative day 14, and 15 features on the day of drainage tube removal (feature labels are in Multimedia Appendix 4). Based on feature dimensions of the patients, we constructed a MultiStep Transformer model with 4 feature prediction modules.

Given the limited sample size (287 patients) and single-center design, we adopted a 5-fold cross-validation scheme for internal validation. The entire cohort was randomly partitioned into 5 approximately equal subsets. In each iteration, one subset was held out as the validation (test) set, and the remaining 4 subsets were used for model training. This process was repeated 5 times so that each subset served as the validation set exactly once. Model performance metrics were averaged across the 5 folds and reported together with their SDs, providing an estimate of variability and robustness. The outcome labels were not evenly distributed across classes. To mitigate the impact of class imbalance, we ensured that the proportion of outcome categories in each fold of the cross-validation was approximately consistent with that of the full cohort by using stratified splitting according to the target labels. In addition, class weights inversely proportional to class frequencies in the training data were applied in the loss function to reduce bias toward the majority class. This strategy helped the model to learn more balanced decision boundaries across different outcome categories.

We calculated the confusion matrices of the results to obtain accuracy, precision, recall, and *F*_1_-score. Regarding the prediction results, actual labels were defined as follows: true positive (TP), positive labels correctly predicted; false positive (FP), negative labels incorrectly predicted; true negative (TN), negative labels correctly predicted; false negative (FN), positive labels incorrectly predicted. The evaluation metrics were calculated as follows:



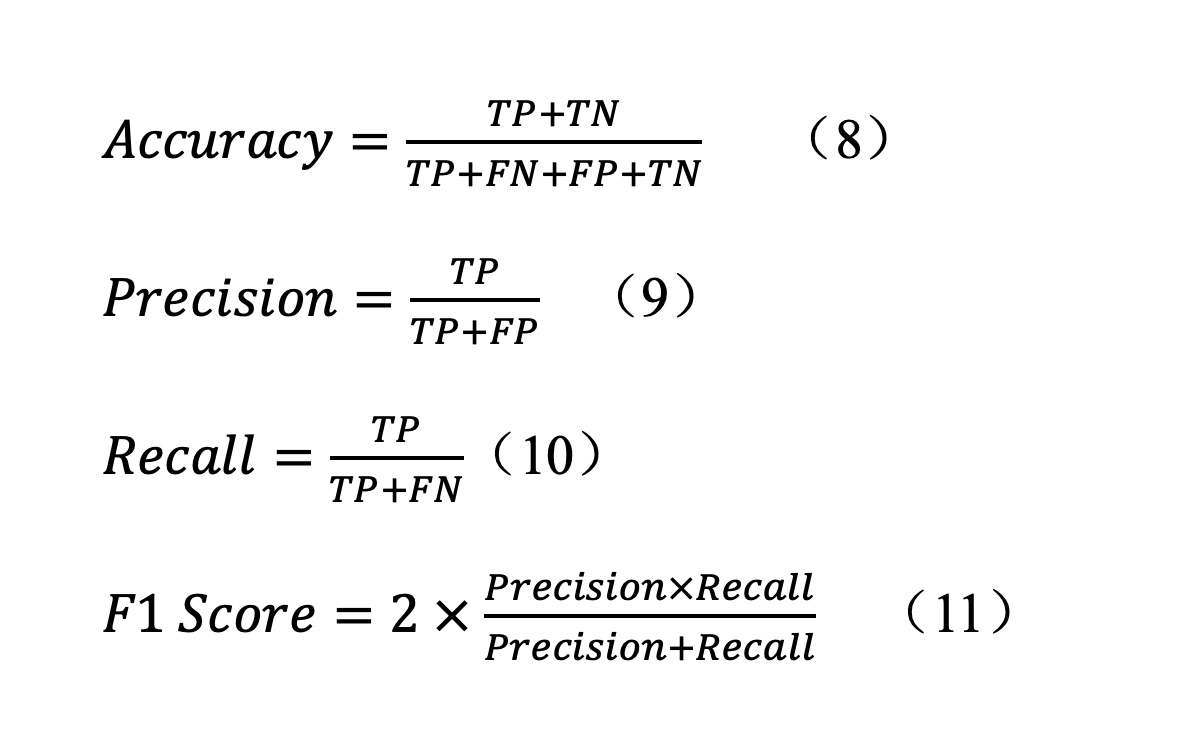



For comparison, we selected the commonly used deep learning models, including temporal convolutional network (TCN), gated recurrent unit (GRU), and transformer. Table 1 presents the comparison of prediction performance between the MultiStep Transformer model and each deep learning model.

**Table 1:** Comparison of model performance.

Model and scene		Accuracy (%), mean (SD)	Precision (%), mean (SD)		Recall (%), mean (SD)		*F*_1_-score (%), mean (SD)	
GRU^a^								
	live_30	77.34 (28.89)	100 (0)		75.66 (30.88)		81.17 (28.30)	
	live_180	66.5 (27.68)	98.1 (0.99)		63.39 (31.91)		70.24 (33.74)	
	heal_180	73.70 (7.20)	75.61 (6.44)		71.92 (10.52)		73.49 (8.05)	
TCN^b^								
	live_30	85.55 (19.8)	100 (0)		84.49 (21.23)		89.83 (15.30)	
	live_180	74.04 (19)	97.92 (2.55)		71.57 (21.23)		80.96 (15.30)	
	heal_180	74.68 (5.86)	77.09 (2.51)		71.94 (12.72)		73.90 (8.03)	
Transformer								
	live_30	88.83 (12.53)	100 (0)		88 (13.40)		93.01 (8.50)	
	live_180	76.33 (15.52)	98.18 (2.28)		74.16 (16.59)		83.54 (12.12)	
	heal_180	78.95 (5.91)	81.75 (3.65)		76.43 (13.4)		78.28 (7.81)	
MultiStepTransformer (Ours)								
	live_30	92.43 (1.68)	96.47 (2.15)		95.37 (1.83)		95.89 (0.92)	
	live_180	88.80 (3.24)	93.99 (1.68)		93.22 (3.90)		93.55 (1.20)	
	heal_180	80.13 (3.68)	81.16 (2.13)		82.02 (6.05)		81.42 (2.82)	

^a^GRU: gated recurrent unit.

^b^TCN: temporal convolutional network.

The proposed method achieved the best performance in terms of accuracy, recall, and *F*_1_-score for the 3 categories of outcomes. The accuracy of the MultiStep Transformer model was 92.43 (1.68) for 30-day survival, 88.80 (3.24) for 180-day survival, and 80.13 (3.68) for 180-day favorable functional outcome, respectively.

Furthermore, we separated the 4 scores (GCS, NIHSS, ICH score, and FUNC score) from the data to predict the three categories of outcomes. The FUNC score is associated with the volume of hematoma, but there were some missing data, and it could not be combined with the other scores for classification. We compared the single-score classification and the combined classification of scores (ICH score, GCS, and NIHSS), respectively. We also used support vector machine (for classification). Using the same 5-fold cross validation, their confusion matrices were calculated to obtain the corresponding indicators (Table 2).

As can be seen, the NIHSS predicts the best results of the traditional scores for 30-day survival. The FUNC score was superior to the other 2 scores in the accurate classification of 180-day survival and 180-day favorable functional outcome. It significantly improved the prediction of 180-day favorable functional outcome, indicating that the FUNC score was better at predicting the long-term prognosis of patients. The combination of the ICH score, GCS, and NIHSS features significantly improved 30-day survival prediction, but the FUNC score still exhibited some advantages in long-term prediction.

**Figure 3:**
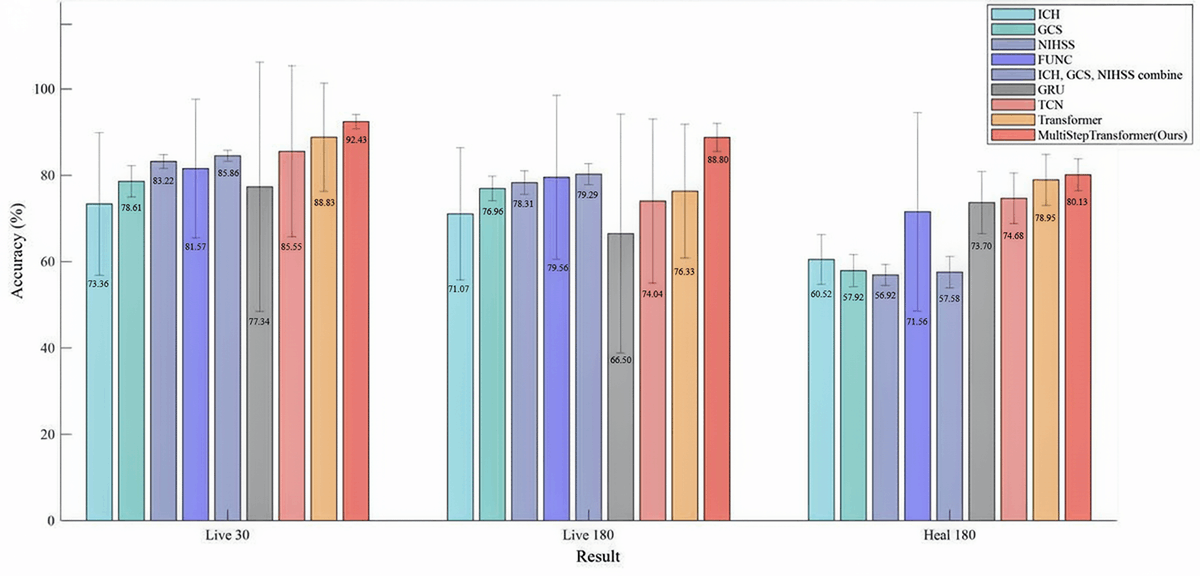
Comparison of classification accuracy across models and traditional scoring methods. FUNC: functional outcome in patients with primary intracerebral hemorrhage; GCS: Glasgow Coma Scale; GRU: gated recurrent unit; ICH: intracerebral hemorrhage; NIHSS: National Institutes of Health Stroke Scale; TCN: temporal convolutional network.

Figure 3 presents a comparison of classification accuracy between the MultiStep Transformer model, traditional scoring methods, and other deep learning models. The results indicated that compared to either traditional scoring methods or other deep learning models, our proposed method offers superior performance in both short-term and long-term predictions of the 3 outcome categories. Furthermore, the MultiStep Transformer model yielded the smallest SD (eg, mean 92.43, SD 1.68 for accuracy in 30-day survival prediction), reflecting its consistent performance across different data subsets, enhanced stability, and strong generalization capability.

**Figure 4:**
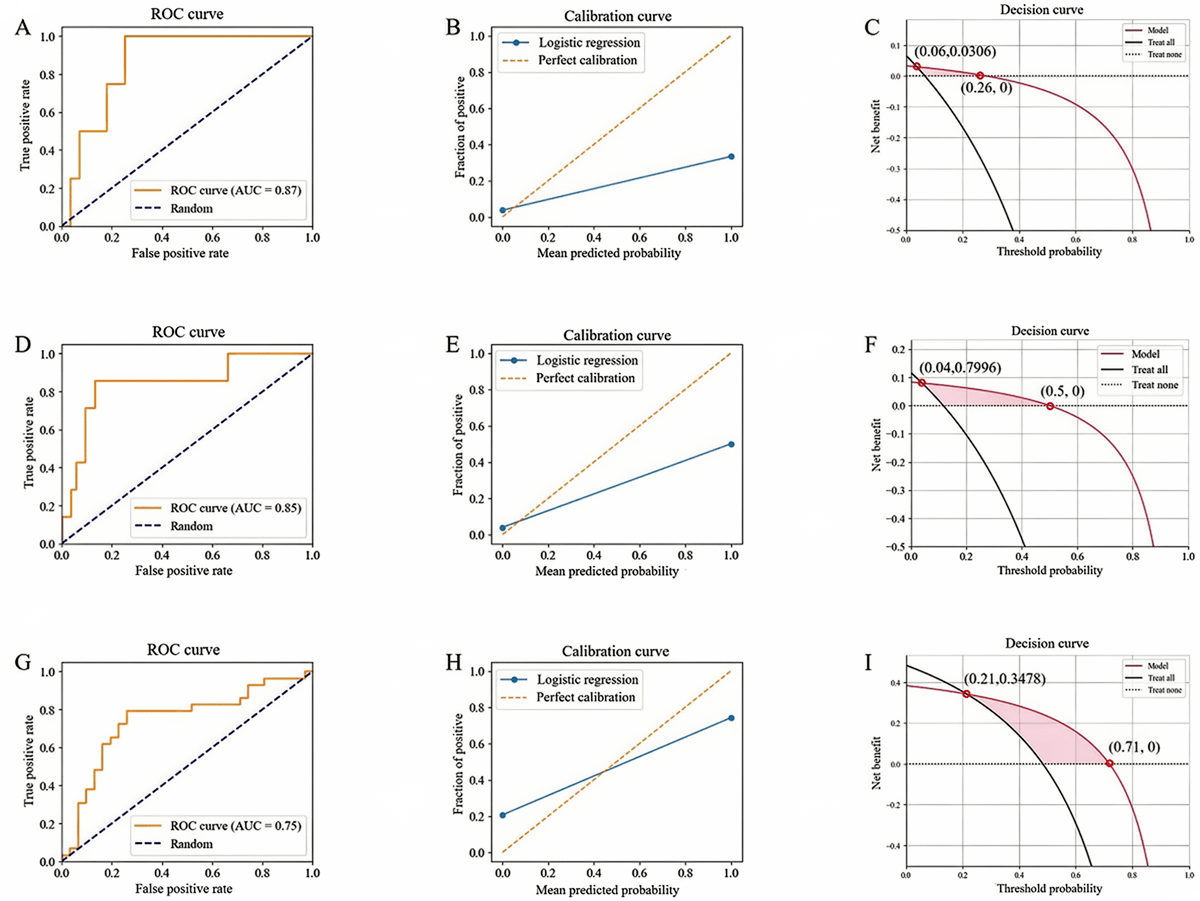
Results of 5‑fold cross‑validation: Panels A-C depict the ROC curve, calibration curve, and decision curve for 30‑day survival, respectively; panels D-F represent those for 180‑day survival; and panels G-I denote those for 180‑day favorable functional outcome. AUC: area under the curve; ROC: receiver operating characteristic.

Figures 4A, D, and G present the receiver operating characteristic (ROC) curves of the model for different outcomes, namely 30-day survival, 180-day survival, and 180-day favorable functional outcome. The model achieved AUROCs of 0.87 (95% CI 0.82-0.92), 0.85 (95% CI 0.77-0.93), and 0.75 (95% CI 0.72-0.78) for these 3 outcomes, respectively.

Furthermore, the calibration curves demonstrated good calibration performance of the model (Figures 4B, E, and H), with corresponding Brier scores of 0.1041, 0.1115, and 0.231, respectively. DCA indicated that the model yielded a net benefit when the threshold probabilities ranged within 0.06-0.26, 0.04-0.5, and 0.21-0.71, respectively (Figures 4C, F, and I). The above findings confirm that this model exhibits favorable generalizability.

**Table 2:** Comparison of traditional scores.

Score and scene		Accuracy (%), mean (SD)	Precision (%), mean (SD)	Recall (%), mean (SD)	*F*_1_-score (%), mean (SD)
ICH^a^ score					
	live_30	73.36 (16.52)	56.56 (4.66)	66.00 (11.4)	54.22 (10.68)
	live_180	71.07 (15.30)	55.96 (4.90)	58.51 (7.03)	53.88 (9.33)
	heal_180	60.52 (5.78)	66.05 (6.37)	59.67 (3.89)	55.65 (5.53)
GCS^b^					
	live_30	78.61 (3.63)	55.27 (5.30)	63.4 (12.93)	55.47 (6.98)
	live_180	76.96 (2.85)	57.64 (7.18)	61.71 (8.53)	57.92 (8.23)
	heal_180	57.92 (3.74)	58.73 (4.62)	56.89 (4.59)	54 (5.61)
NIHSS^c^					
	live_30	83.22 (1.60)	58.03 (4.79)	67.72 (11.77)	59.60 (6)
	live_180	78.31 (2.72)	55.88 (7.82)	61.67 (11.79)	56.94 (9.08)
	heal_180	56.92 (2.45)	57.43 (4.79)	55.31 (3.29)	52.36 (4.51)
FUNC^d^ score					
	live_30	81.57 (16.08)	70 (24.81)	66.58 (27.95)	67.46 (26.83)
	live_180	79.56 (18.99)	68.75 (25.62)	65.75 (28.37)	65.99 (27.79)
	heal_180	71.56 (23.01)	74.40 (20.49)	70.83 (19.43)	68.10 (23.25)
Combination of the ICH score, GCS, and NIHSS					
	live_30	85.86 (1.61)	60.67 (6.36)	70.98 (13.28)	62.93 (8.07)
	live_180	79.29 (3.12)	58.39 (5.30)	63.17 (10.61)	59.02 (6.64)
	heal_180	57.58 (3.75)	57.37 (6.33)	56.00 (5.09)	52.91 (6.86)
MultiStep Transformer (ours)					
	live_30	92.43 (1.68)	96.47 (2.15)	95.37 (1.83)	95.89 (0.92)
	live_180	88.80 (3.24)	93.99 (1.68)	93.22 (3.90)	93.55 (1.20)
	heal_180	80.13 (3.68)	81.16 (2.13)	82.02 (6.05)	81.42 (2.82)

^a^ICH: intracerebral hemorrhage.

^b^GCS: Glasgow Coma Scale.

^c^NIHSS: National Institutes of Health Stroke Scale.

^d^FUNC: functional outcome in patients with primary intracerebral hemorrhage.

### Model Interpretation

In this study, we used deep neural networks to classify clinical data for predicting patients’ outcomes. Specifically, we visualized the attribution values calculated by the model alongside the corresponding input feature values in the same diagram to interpret the model’s decision-making mechanism.

**Figure 5:**
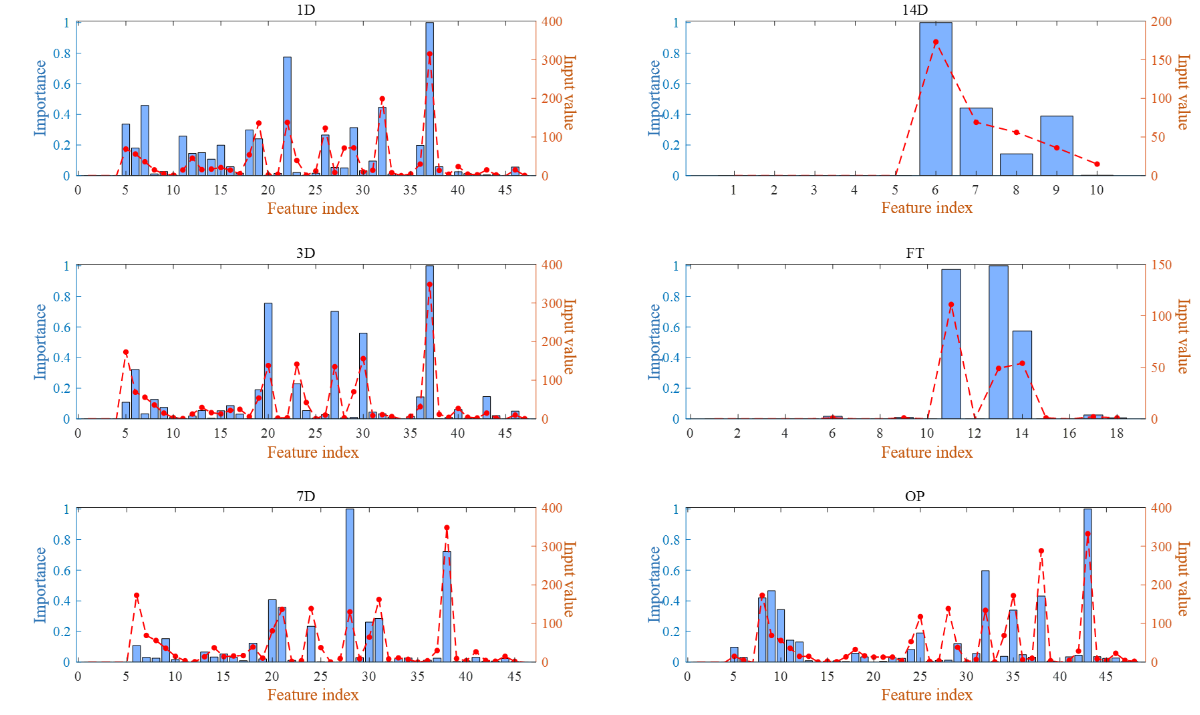
Visualization of attribution values of features.

The attribution values indicated the contribution of each clinical feature to the model’s output. The feature importance was relatively similar between postoperative day 1 and day 3, while some changes emerged in the key clinical features affecting predictions by days 7 and 14 (Figure 5).

The horizontal axis in Figure 5 represents the indices of the input features as discrete integers, facilitating the identification of specific features. The left vertical axis denotes the attribution values, displayed with blue bars that illustrate each feature’s relative contribution to the output. The right vertical axis corresponds to the original feature values, plotted as a red dashed line to visualize the correspondence between attributions and raw data. This integrated plot helps identify the most influential features on the model’s predictions, enabling a more comprehensive understanding of the model’s behavior.

## Discussion

### Principal Findings

This study reports the construction process and model performance of the MultiStep Transformer model, a novel deep learning model that can dynamically predict short-term and long-term survival and functional outcome in patients with ICH undergoing MIS. The experimental results showed that the model significantly outperformed the mainstream deep learning models (GRU, TCN, and transformer) and traditional clinical scores (GCS, NIHSS, ICH score, and FUNC score) on the 3 prediction tasks of 30-day survival, 180-day survival, and 180-day favorable functional outcome.

In patients undergoing MIS for ICH, neurological functions, vital signs, imaging findings, and laboratory parameters are subjected to continuous changes, generating substantial clinical data. However, temporal data in EHR present challenges, including irregular recording intervals and variable sequence lengths across individuals. Meanwhile, most of the previous studies have conducted predictions at fixed time points, whereas many clinical scenarios necessitate continuous outcome updates with the emergence of new data [22]. Therefore, it is essential to develop a dynamic model to process variably dimensional features. This approach not only addresses the needs of clinical practice but also allows personalized management of patients.

The MultiStep Transformer model incorporates innovative designs to address common challenges in real-world clinical data, such as missing time points, heterogeneous feature dimensions, and inconsistent sampling frequencies.

First, multistage feature prediction addresses data missingness. Through layered stepwise linear mapping, the model not only imputes missing values at certain time points but also enhances temporal dependencies among features, thereby improving the accuracy of predictions.

Second, time point–specific embedding resolves dimensionality inconsistency. Independent linear embedding layers ensure unified representation of heterogeneous input data, strengthening the generalizability of the model.

Third, the self-attention mechanism of the transformer captures long-range dependencies. Compared to traditional recurrent neural networks, the transformer architecture more effectively learns complex nonlinear dependencies and interactions across several time points.

Finally, we visualized feature importance through attribution values. The values revealed which clinical features played a pivotal role in the final predictions. A higher attribution value of a feature suggested a greater influence on the model’s decision-making. The MultiStep Transformer model successfully captured the dynamic shifts of key perioperative features during MIS, thereby enhancing the interpretability of the model and its credibility in clinical practice.

### Comparison With Prior Work

TCN is a feedforward network architecture that models time series by stacking convolutional layers [23]. Under the constraint of convolutional layers, it can effectively prevent information leakage. Multilayer convolution can also capture local time dependence and short-term dynamic changes in multi–time point clinical data. GRU is a type of recurrent neural network, which uses a gating unit to control the memory and forgetting of information. It has a relatively small number of parameters and high training stability and can model the dependence from local to global. For clinical data with multiple time points, GRU can better extract cross-temporal global features [24]. With the attention mechanism as the core, Transformer can model the relationships between any time points in a sequence in a global scope [25], making it suitable for dealing with long sequences and complex temporal patterns. In addition, it has good parallel computing ability and can effectively use global information.

Compared to the mainstream deep learning models, the MultiStep Transformer model offers full usage and efficient modeling of complex imbalanced time-series data by introducing a multistage feature cycle prediction module, time-sharing embedding mechanism, and transformer encoder structure. Besides, the modular design of the MultiStep Transformer model enables adaptation to flexible inputs, supports dynamic input time points, and is suitable for clinical scenarios with different monitoring frequencies, thereby improving its practicability.

In 30-day survival prediction, GRU, TCN, and transformer achieved 100% precision, indicating rare false positive errors, but their low recall suggests many missed diagnoses. The MultiStep Transformer exhibited a significant improvement in recall and *F*_1_-score, suggesting a more accurate identification ability while avoiding excessive conservatism. The performance of all models decreased with the prolongation of prediction time, but the MultiStep Transformer still showed a high *F*_1_-score in 180-day survival prediction, with greatly improved recall. These findings imply that it is more sensitive in long-term prediction and not overly conservative. In the prediction of a favorable functional outcome at 180 days, all models performed worse compared to their performance in survival prediction, highlighting the challenges in predicting functional outcomes. The MultiStep Transformer was the only model with a recall of more than 80%, indicating that it was more advantageous in identifying patients with a greater chance of recovery.

Traditional scoring systems are widely used in clinical practice due to their simplicity and have demonstrated considerable value in predicting the outcomes of ICH. For predicting mortality, the ICH score achieved AUROCs of 0.83-0.84, while the FUNC score showed AUROCs of 0.79-0.83. In predicting functional outcomes, the ICH score attained AUROCs of 0.77-0.82, compared to 0.76-0.78 for the FUNC score [26]. When predicting death or severe disability, the NIHSS score yielded an AUROC of 0.796, the GCS score achieved an AUROC of 0.650, and the ICH score revealed an AUROC of 0.674 [27]. By comparison, the model developed in this study demonstrated superior predictive performance. For the prediction of 30‑day survival, 180‑day survival, and 180‑day favorable functional outcome, the model achieved AUROCs of 0.87 (95% CI 0.82-0.92), 0.85 (95% CI 0.77-0.93), and 0.75 (95% CI 0.72-0.78), respectively. Meanwhile, calibration curves and DCA confirmed that this model has excellent calibration performance and definite clinical benefit. Furthermore, the inherent limitations of traditional scoring systems are becoming increasingly apparent. McCracken et al. indicated that nearly two decades after its introduction, the predictive accuracy of the ICH score may no longer be sufficient for contemporary clinical practice, necessitating adjustments and optimization [28]. More importantly, the clinical guideline “Guidelines for Neuroprognostication in Critically Ill Adults with Intracerebral Hemorrhage” emphasizes that no single score or clinical variable should be used as the sole basis for prognostic judgment at 3 months or beyond [29]. Accordingly, factors such as preoperative functional status, surgical complications, and intervention timing should be comprehensively considered when developing assessment tools for patients undergoing surgery [30]. The dynamic prognostic model developed in this study aligns with this philosophy. From a clinical perspective, accurate prediction of long-term functional outcomes is crucial for rehabilitation planning and patient communication. For predicting 180-day functional outcomes, the most challenging task, our model achieved an *F*_1_-score of 81.42%, significantly surpassing traditional scoring systems (maximum 71.56%), suggesting its substantial potential in guiding rehabilitation strategies.

### Limitations

There are some limitations to this study. First, this study was a single-center retrospective study with a small sample size, which constitutes the most critical methodological limitation. Population heterogeneity, treatment variability, and fluctuations in data quality may potentially compromise model performance. Although our dataset, spanning over ten years and including medical records from both local and nonlocal patients, confers a certain degree of representativeness, and despite rigorous cross-validation for model performance evaluation, single-center data are inevitably influenced by institutional clinical protocols and inherent biases of retrospective data collection. This poses a distinct challenge for dynamic models, whose generalizability is limited by institutional variations in feature distributions and missing patterns. Second, the puncture needle used in MIS leads to artifacts in CT images, which will affect the accurate calculation of hematoma volume. Third, the model only predicted binary outcomes, such as survival or death, favorable prognosis, and unfavorable prognosis, but could not predict the specific mRS score. The prediction ability of the functional outcome was not comprehensive enough. Finally, we collected clinical data at a few time points, which may have affected the performance of the model. In the future, more clinical data at more time points can help train the model more effectively.

### Future Work

All features incorporated into this model are derived from routine clinical practice in MIS for ICH, eliminating the need for additional data collection efforts. Meanwhile, the model supports continuous integration of the latest data and dynamic updating of prediction outcomes, which can precisely match the time-varying characteristics of ICH progression and the clinical decision-making needs of MIS. Leveraging these advantages, the ideal application of the dynamic prediction model developed in this study is to be embedded in the EHR system, thereby providing clinicians with real-time individualized prognostic predictions to support accurate decision-making. However, its implementation faces obstacles such as technical integration and workflow adaptation. Future work will advance in multiple directions. First, more time-point data will be incorporated to improve the model’s performance, and external validation of the model will be conducted in multicenter, prospective cohorts. This is a key step to evaluate its real-world generalizability and promote clinical translation. Second, standardized interfaces will be developed to achieve seamless integration of the model with different EHR systems and ensure real-time data updates. Third, the actual impact of the model on clinical decision-making and final clinical outcomes will be evaluated in real clinical settings, and a replicable workflow solution will be formulated.

### Conclusions

Based on clinical data, the MultiStep Transformer model uses deep learning methods to achieve the goal of dynamic, comprehensive, and accurate prediction. It can stably output multiple prediction results in a single prognostic model at the same time. This study explored the individualized management of patients with ICH undergoing MIS, providing a new method for predicting short-term and long-term survival and functional outcomes and bringing a reference for the diagnosis and treatment of ICH in clinical practice.

## Appendix

Multimedia Appendix 1 Baseline Characteristics of Patients Classified by 30-day Survival or Mortality.

Multimedia Appendix 2 Baseline Characteristics of Patients Classified by 180-day Survival or Mortality.

Multimedia Appendix 3 Baseline Characteristics of Patients Classified by 180-day Favorable or Unfavorable Functional Outcome.

Multimedia Appendix 4 Feature labels.

## Abbreviations

AUROC: area under the receiver operating characteristic curve
CT: computer tomography
DCA: decision curve analysis
EHR: electronic health record
ENRICH: Early Minimally Invasive Removal of Intracerebral Hemorrhage
FN: false negative
FP: false positive
FUNC: Functional Outcome in Patients with Primary Intracerebral Hemorrhage
GCS: Glasgow Coma Scale
GRU: gated recurrent unit
ICH: intracerebral hemorrhage
MIS: minimally invasive surgery
MISTIE Ⅲ: Minimally Invasive Surgery Plus Alteplase for Intracerebral Hemorrhage Evacuation Phase 3
mRS: modified Rankin Scale
NIHSS: National Institute of Health Stroke Scale
ROC: receiver operating characteristic
STICH: Surgical Trial in Intracerebral Haemorrhage
STICH II: Surgical Trial in Intracerebral Haemorrhage II
TCN: temporal convolutional network
TN: true negative
TP: true positive
TRIPOD-AI: Transparent Reporting of a multivariable prediction model for Individual Prognosis Or Diagnosis–Artificial Intelligence

## Data Availability

The datasets used during the current study are available from the corresponding author upon reasonable request.
