# Supplementary material for: Deep Learning for Dynamic Prognostic Prediction in Minimally Invasive Surgery for Intracerebral Hemorrhage: Model Development and Validation Study
Source: JMIR Med Inform. 2026 Jan 7;14:e86327. doi: 10.2196/86327 (PMC12824578; doi:10.2196/86327)
Supplement: Multimedia Appendix 1 [file medinform_v14i1e86327_app1.docx]

Table S1. Baseline Characteristics of Patients Classified by 30-day Survival or Mortality.

| Variable | 30-day Survival | 30-day Mortality |  |
| --- | --- | --- | --- |
|  | N = 265 | N = 22 | *p-value* |
| **Demographics** |  |  |  |
| Men, n (%) | 179 (67.5) | 16 (72.7) | 0.617 |
| Age (years), median (IQR) | 53.0 (45.0, 61.0) | 56.5 (50.0, 62.0) | 0.277 |
| **Past Medical History, n (%)** |  |  |  |
| Smoking | 26 (9.81) | 6 (27.3) | 0.024 |
| Hypertension | 106 (40.0) | 9 (40.9) | 0.933 |
| Antiplatelet/Anticoagulant Therapy | 6 (2.26) | 1 (4.55) | 0.431 |
| **Hematoma Status** |  |  |  |
| Hematoma Volume (mL), median (IQR) | 30.6 (23.2, 44.6) | 42.8 (25.7, 62.2) | 0.063 |
| Intraventricular Hemorrhage, n (%) | 109 (41.1) | 15 (68.2) | 0.014 |
| **Cerebrovascular Status, n (%)** |  |  |  |
| Cerebral Arteriosclerosis | 125 (47.2) | 9 (40.9) | 0.572 |
| Cerebral Vascular Stenosis | 26 (9.81) | 3 (13.6) | 0.475 |
| Cerebral Amyloid Angiopathy | 15 (5.66) | 4 (18.2) | 0.047 |
| **Vital Signs, median (IQR)** |  |  |  |
| Systolic Blood Pressure (mmHg) | 158.0 (141.0, 171.0) | 167.5 (159.0, 178.0) | 0.009 |
| Diastolic Blood Pressure (mmHg) | 93.0 (81.0, 105.0) | 87.0 (84.0, 106.0) | 0.829 |
| Pulse Rate (/min) | 80.0 (70.0, 91.0) | 84.0 (77.0, 100.0) | 0.197 |
| Body Temperature (℃) | 36.7 (36.3, 37.2) | 36.5 (36.2, 37.1) | 0.143 |
| Respiratory Rate (/min) | 18.0 (16.0, 20.0) | 18.5 (17.0, 20.0) | 0.113 |
| **Neurological Score, median (IQR)** |  |  |  |
| GCS | 12.0 (10.0, 14.0) | 8.0 (6.0, 12.0) | 0.001 |
| NIHSS | 12.0 (10.0, 14.0) | 25.0 (11.0, 35.0) | 0.014 |
| ICH Score | 1.0 (0.0, 1.0) | 1.5 (1.0, 2.0) | <0.001 |
| FUNC Score | 9.0 (8.0, 10.0) | 8.0 (6.0, 9.0) | 0.005 |
| **Laboratory Studies, median (IQR)** |  |  |  |
| Fibrinogen (g/L) | 4.0 (3.3, 5.1) | 4.5 (2.7, 5.4) | 0.857 |
| International Normalized Ratio (INR) | 1.0 (1.0, 1.1) | 1.0 (1.0, 1.1) | 0.439 |
| Prothrombin Time (second) | 13.5 (12.9, 14.0) | 13.5 (12.9, 14.0) | 0.644 |
| Activated Partial Thromboplastin Time, (second) | 34.3 (32.4, 37.0) | 35.0 (33.6, 36.5) | 0.624 |
| Thrombin Time, (second) | 16.1 (15.3, 16.9) | 16.4 (15.1, 16.9) | 0.706 |
| Alanine Aminotransferase, (U/L) | 17.0 (12.0, 26.0) | 21.0 (11.0, 41.0) | 0.424 |
| Aspartate Aminotransferase, (U/L) | 20.0 (16.0, 28.0) | 22.0 (17.0, 34.0) | 0.523 |
| Total Bilirubin, (umol/L) | 11.4 (7.7, 15.6) | 9.9 (6.9, 13.7) | 0.248 |
| Direct Bilirubin, (umol/L) | 3.7 (2.6, 5.2) | 4.1 (1.9, 5.0) | 0.785 |
| Serum Creatinine, (umol/L) | 66.0 (53.0, 82.0) | 69.0 (56.0, 94.0) | 0.322 |
| Serum Uric Acid, (umol/L) | 200.0 (137.0, 270.0) | 256.8 (127.0, 377.0) | 0.283 |
| Serum Total Calcium, (mmol/L) | 2.3 (2.2, 2.3) | 2.3 (2.1, 2.4) | 0.699 |
| Serum Potassium, (mmol/L) | 3.8 (3.5, 4.1) | 3.7 (3.4, 4.0) | 0.626 |
| Serum Sodium, (mmol/L) | 134.3 (1.4, 139.7) | 134.2 (1.5, 141.6) | 0.540 |
| Serum Albumin, (g/L) | 39.9 (35.8, 43.7) | 38.2 (34.0, 45.5) | 0.908 |
| Lymphocyte Count, (*10^9/L) | 1.0 (0.7, 1.4) | 0.8 (0.6, 1.0) | 0.033 |
| White Blood Cell Count, (*10^9/L) | 10.4 (8.6, 12.7) | 13.2 (10.1, 16.4) | 0.006 |
| Hemoglobin, (g/L) | 137.0 (125.0, 149.0) | 137.5 (116.0, 146.0) | 0.393 |
| D-Dimer, (ug/mL FEU) | 1.0 (0.5, 2.0) | 1.5 (0.7, 2.0) | 0.144 |
| Total Protein, (g/L) | 71.2 (66.0, 75.0) | 72.2 (65.9, 78.6) | 0.387 |
| Red Blood Cell Count, (*10^12/L) | 4.5 (4.1, 4.9) | 4.5 (3.9, 4.8) | 0.481 |
| Platelet Count, (*10^9/L) | 187.0 (147.0, 230.0) | 181.0 (138.0, 217.0) | 0.425 |
| Neutrophil Count, (*10^9/L) | 8.6 (6.7, 10.6) | 11.3 (8.7, 14.6) | 0.003 |
| Gamma-Glutamyl Transferase, (U/L) | 27.0 (18.0, 47.0) | 23.5 (14.0, 56.0) | 0.483 |
| Lactate Dehydrogenase, (U/L) | 230.0 (193.0, 272.0) | 220.5 (200.0, 273.0) | 0.933 |
| Monocyte Percentage, n (%) | 5.9 (4.5, 7.4) | 5.2 (3.4, 6.0) | 0.025 |
| Basophil Percentage, n (%) | 0.1 (0.1, 0.2) | 0.1 (0.1, 0.2) | 0.199 |
| Urea, (mmol/L) | 5.6 (4.2, 7.5) | 5.6 (4.4, 17.2) | 0.200 |
| Mean Corpuscular Hemoglobin, (pg) | 30.0 (28.9, 31.1) | 30.1 (29.4, 31.0) | 0.743 |
| Mean Corpuscular Hemoglobin Concentration, (g/L) | 333.0 (322.0, 340.0) | 334.5 (316.0, 342.0) | 0.742 |
| Mean Platelet Volume, (fL) | 10.7 (10.1, 11.7) | 11.1 (9.7, 12.6) | 0.435 |
| Total Cholesterol, (mmol/L) | 4.2 (3.6, 5.0) | 4.0 (3.2, 4.2) | 0.123 |
