# Supplementary material for: Deep Learning for Dynamic Prognostic Prediction in Minimally Invasive Surgery for Intracerebral Hemorrhage: Model Development and Validation Study
Source: JMIR Med Inform. 2026 Jan 7;14:e86327. doi: 10.2196/86327 (PMC12824578; doi:10.2196/86327)
Supplement: Multimedia Appendix 2 [file medinform_v14i1e86327_app2.docx]

Table S2. Baseline Characteristics of Patients Classified by 180-day Survival or Mortality.

| Variable | 180-day Survival | 180-day Mortality |  |
| --- | --- | --- | --- |
|  | N = 252 | N = 35 | *p-value* |
| **Demographics** |  |  |  |
| Men, n (%) | 170 (67.5) | 25 (71.4) | 0.637 |
| Age (years), median (IQR) | 52.0 (45.0, 61.0) | 56.0 (50.0, 65.0) | 0.060 |
| **Past Medical History, n (%)** |  |  |  |
| Smoking | 26 (10.3) | 6 (17.1) | 0.250 |
| Hypertension | 100 (39.7) | 15 (42.9) | 0.719 |
| Antiplatelet/Anticoagulant Therapy | 6 (2.38) | 1 (2.86) | 0.602 |
| **Hematoma Status** |  |  |  |
| Hematoma Volume (mL), median (IQR) | 30.6 (23.2, 44.6) | 39.9 (23.1, 58.8) | 0.086 |
| Intraventricular Hemorrhage, n (%) | 103 (40.9) | 21 (60.0) | 0.032 |
| **Cerebrovascular Status, n (%)** |  |  |  |
| Cerebral Arteriosclerosis | 121 (48.0) | 13 (37.1) | 0.227 |
| Cerebral Vascular Stenosis | 25 (9.92) | 4 (11.4) | 0.765 |
| Cerebral Amyloid Angiopathy | 15 (5.95) | 4 (11.4) | 0.266 |
| **Vital Signs, median (IQR)** |  |  |  |
| Systolic Blood Pressure (mmHg) | 158.0 (141.0, 171.5) | 164.0 (150.0, 176.0) | 0.069 |
| Diastolic Blood Pressure (mmHg) | 92.0 (81.0, 105.0) | 94.0 (85.0, 106.0) | 0.625 |
| Pulse Rate (/min) | 80.0 (70.0, 91.0) | 84.0 (71.0, 100.0) | 0.135 |
| Body Temperature (℃) | 36.7 (36.3, 37.2) | 36.5 (36.2, 37.2) | 0.134 |
| Respiratory Rate (/min) | 18.0 (16.0, 20.0) | 18.0 (16.0, 20.0) | 0.323 |
| **Neurological Score, median (IQR)** |  |  |  |
| GCS | 13.0 (10.0, 14.0) | 11.0 (6.0, 13.0) | 0.001 |
| NIHSS | 12.0 (10.0, 14.0) | 14.0 (10.0, 35.0) | 0.034 |
| ICH Score | 1.0 (0.0, 1.0) | 1.0 (1.0, 2.0) | 0.004 |
| FUNC Score | 9.0 (8.0, 10.0) | 8.0 (6.0, 10.0) | 0.003 |
| **Laboratory Studies, median (IQR)** |  |  |  |
| Fibrinogen (g/L) | 4.0 (3.3, 5.2) | 4.2 (2.8, 5.0) | 0.571 |
| International Normalized Ratio (INR) | 1.0 (1.0, 1.1) | 1.0 (1.0, 1.1) | 0.692 |
| Prothrombin Time (second) | 13.5 (12.9, 14.0) | 13.4 (12.9, 14.0) | 0.894 |
| Activated Partial Thromboplastin Time, (second) | 34.3 (32.4, 37.1) | 34.8 (32.9, 36.6) | 0.811 |
| Thrombin Time, (second) | 16.1 (15.2, 16.9) | 16.3 (15.3, 16.7) | 0.804 |
| Alanine Aminotransferase, (U/L) | 17.0 (12.0, 26.0) | 20.0 (11.0, 28.0) | 0.490 |
| Aspartate Aminotransferase, (U/L) | 20.0 (16.0, 28.0) | 23.0 (19.0, 33.0) | 0.147 |
| Total Bilirubin, (umol/L) | 11.3 (7.7, 15.7) | 10.4 (7.1, 14.1) | 0.270 |
| Direct Bilirubin, (umol/L) | 3.7 (2.6, 5.2) | 3.8 (2.2, 4.9) | 0.748 |
| Serum Creatinine, (umol/L) | 66.0 (53.0, 81.0) | 69.0 (56.0, 94.0) | 0.083 |
| Serum Uric Acid, (umol/L) | 200.0 (134.3, 267.5) | 286.0 (144.0, 387.0) | 0.086 |
| Serum Total Calcium, (mmol/L) | 2.2 (2.2, 2.3) | 2.3 (2.2, 2.4) | 0.132 |
| Serum Potassium, (mmol/L) | 3.8 (3.4, 4.1) | 3.8 (3.5, 4.2) | 0.422 |
| Serum Sodium, (mmol/L) | 134.4 (1.4, 139.7) | 134.0 (1.4, 141.6) | 0.748 |
| Serum Albumin, (g/L) | 39.8 (35.5, 43.7) | 40.3 (36.4, 45.5) | 0.288 |
| Lymphocyte Count, (*10^9/L) | 1.0 (0.7, 1.4) | 0.8 (0.6, 1.1) | 0.043 |
| White Blood Cell Count, (*10^9/L) | 10.4 (8.6, 12.7) | 11.9 (9.9, 14.6) | 0.006 |
| Hemoglobin, (g/L) | 137.0 (125.0, 150.0) | 134.0 (122.0, 145.0) | 0.190 |
| D-Dimer, (ug/mL FEU) | 1.0 (0.5, 2.0) | 1.2 (0.6, 2.3) | 0.224 |
| Total Protein, (g/L) | 71.0 (65.9, 75.0) | 72.5 (67.8, 79.8) | 0.081 |
| Red Blood Cell Count, (*10^12/L) | 4.5 (4.1, 4.9) | 4.3 (4.0, 4.7) | 0.127 |
| Platelet Count, (*10^9/L) | 187.0 (146.0, 229.5) | 184.0 (138.0, 229.0) | 0.818 |
| Neutrophil Count, (*10^9/L) | 8.6 (6.6, 10.6) | 9.7 (8.3, 13.1) | 0.002 |
| Gamma-Glutamyl Transferase, (U/L) | 27.0 (18.0, 47.0) | 25.0 (15.0, 63.0) | 0.705 |
| Lactate Dehydrogenase, (U/L) | 230.0 (192.5, 273.0) | 225.0 (200.0, 272.0) | 0.934 |
| Monocyte Percentage, n (%) | 5.9 (4.5, 7.4) | 5.2 (3.9, 6.6) | 0.021 |
| Basophil Percentage, n (%) | 0.1 (0.1, 0.2) | 0.1 (0.1, 0.2) | 0.628 |
| Urea, (mmol/L) | 5.5 (4.1, 7.5) | 6.4 (4.4, 13.4) | 0.078 |
| Mean Corpuscular Hemoglobin, (pg) | 30.0 (28.9, 31.1) | 30.1 (29.4, 31.0) | 0.395 |
| Mean Corpuscular Hemoglobin Concentration, (g/L) | 333.0 (323.0, 340.5) | 333.0 (314.0, 340.0) | 0.233 |
| Mean Platelet Volume, (fL) | 10.8 (10.1, 11.7) | 10.6 (9.4, 12.5) | 0.990 |
| Total Cholesterol, (mmol/L) | 4.2 (3.5, 5.0) | 4.0 (3.6, 4.6) | 0.603 |
