# Supplementary material for: Deep Learning for Dynamic Prognostic Prediction in Minimally Invasive Surgery for Intracerebral Hemorrhage: Model Development and Validation Study
Source: JMIR Med Inform. 2026 Jan 7;14:e86327. doi: 10.2196/86327 (PMC12824578; doi:10.2196/86327)
Supplement: Multimedia Appendix 3 [file medinform_v14i1e86327_app3.docx]

Table S3. Baseline Characteristics of Patients Classified by 180-day Favorable or Unfavorable Functional Outcome.

| Variable | 180-day Favorable Functional Outcome | 180-day Unfavorable Functional Outcome |  |
| --- | --- | --- | --- |
|  | N = 150 | N = 137 | *p-value* |
| **Demographics** |  |  |  |
| Men, n (%) | 100 (66.7) | 95 (69.3) | 0.627 |
| Age (years), median (IQR) | 52.0 (45.0, 60.0) | 53.0 (46.0, 63.0) | 0.112 |
| **Past Medical History, n (%)** |  |  |  |
| Smoking | 15 (10.0) | 17 (12.4) | 0.517 |
| Hypertension | 44 (29.3) | 71 (51.8) | <0.001 |
| Antiplatelet/Anticoagulant Therapy | 5 (3.33) | 2 (1.46) | 0.451 |
| **Hematoma Status** |  |  |  |
| Hematoma Volume (mL), median (IQR) | 28.5 (22.1, 39.5) | 33.9 (25.7, 50.5) | 0.003 |
| Intraventricular Hemorrhage, n (%) | 51 (34.0) | 73 (53.3) | <0.001 |
| **Cerebrovascular Status, n (%)** |  |  |  |
| Cerebral Arteriosclerosis | 78 (52.0) | 56 (40.9) | 0.059 |
| Cerebral Vascular Stenosis | 20 (13.3) | 9 (6.57) | 0.058 |
| Cerebral Amyloid Angiopathy | 7 (4.67) | 12 (8.76) | 0.164 |
| **Vital Signs, median (IQR)** |  |  |  |
| Systolic Blood Pressure (mmHg) | 155.0 (138.0, 170.0) | 163.0 (146.0, 175.0) | 0.029 |
| Diastolic Blood Pressure (mmHg) | 92.0 (81.0, 104.0) | 95.0 (81.0, 107.0) | 0.385 |
| Pulse Rate (/min) | 78.0 (70.0, 91.0) | 82.0 (70.0, 94.0) | 0.160 |
| Body Temperature (℃) | 36.7 (36.3, 37.2) | 36.6 (36.3, 37.2) | 0.954 |
| Respiratory Rate (/min) | 18.0 (16.0, 20.0) | 18.0 (16.0, 20.0) | 0.114 |
| **Neurological Score, median (IQR)** |  |  |  |
| GCS | 13.0 (11.0, 14.0) | 12.0 (7.0, 14.0) | 0.007 |
| NIHSS | 11.0 (10.0, 14.0) | 13.0 (10.0, 18.0) | <0.001 |
| ICH Score | 1.0 (0.0, 1.0) | 1.0 (0.0, 2.0) | 0.003 |
| FUNC Score | 9.5 (8.0, 10.0) | 8.0 (7.0, 10.0) | <0.001 |
| **Laboratory Studies, median (IQR)** |  |  |  |
| Fibrinogen (g/L) | 3.9 (3.3, 4.9) | 4.2 (3.4, 5.2) | 0.264 |
| International Normalized Ratio (INR) | 1.0 (1.0, 1.1) | 1.0 (1.0, 1.1) | 0.864 |
| Prothrombin Time (second) | 13.4 (12.9, 14.0) | 13.5 (12.9, 14.0) | 0.965 |
| Activated Partial Thromboplastin Time, (second) | 34.0 (32.3, 36.4) | 35.2 (32.9, 37.5) | 0.068 |
| Thrombin Time, (second) | 16.2 (15.2, 17.2) | 16.0 (15.3, 16.7) | 0.242 |
| Alanine Aminotransferase, (U/L) | 16.0 (12.0, 24.0) | 17.0 (13.0, 30.0) | 0.091 |
| Aspartate Aminotransferase, (U/L) | 19.0 (16.0, 27.0) | 21.0 (17.0, 29.0) | 0.106 |
| Total Bilirubin, (umol/L) | 11.9 (7.8, 15.6) | 10.6 (7.4, 14.8) | 0.234 |
| Direct Bilirubin, (umol/L) | 3.9 (2.7, 5.3) | 3.6 (2.3, 5.0) | 0.141 |
| Serum Creatinine, (umol/L) | 67.0 (55.0, 82.0) | 66.0 (52.0, 81.0) | 0.803 |
| Serum Uric Acid, (umol/L) | 203.7 (139.0, 271.0) | 190.0 (129.0, 287.0) | 0.606 |
| Serum Total Calcium, (mmol/L) | 2.3 (2.2, 2.3) | 2.2 (2.2, 2.3) | 0.505 |
| Serum Potassium, (mmol/L) | 3.8 (3.5, 4.1) | 3.7 (3.4, 4.0) | 0.362 |
| Serum Sodium, (mmol/L) | 131.7 (1.4, 139.0) | 134.9 (1.4, 140.7) | 0.013 |
| Serum Albumin, (g/L) | 39.9 (36.1, 43.9) | 39.9 (34.7, 43.9) | 0.700 |
| Lymphocyte Count, (*10^9/L) | 1.0 (0.7, 1.4) | 1.0 (0.7, 1.3) | 0.509 |
| White Blood Cell Count, (*10^9/L) | 10.2 (8.2, 12.6) | 10.7 (9.1, 13.4) | 0.079 |
| Hemoglobin, (g/L) | 138.5 (126.0, 150.0) | 134.0 (123.0, 148.0) | 0.362 |
| D-Dimer, (ug/mL FEU) | 0.9 (0.5, 1.7) | 1.0 (0.6, 2.3) | 0.087 |
| Total Protein, (g/L) | 70.7 (65.7, 75.0) | 71.9 (67.4, 76.2) | 0.135 |
| Red Blood Cell Count, (*10^12/L) | 4.6 (4.2, 5.0) | 4.4 (4.1, 4.9) | 0.238 |
| Platelet Count, (*10^9/L) | 194.0 (151.0, 231.0) | 180.0 (142.0, 223.0) | 0.098 |
| Neutrophil Count, (*10^9/L) | 8.2 (6.5, 10.6) | 8.9 (7.2, 11.3) | 0.057 |
| Gamma-Glutamyl Transferase, (U/L) | 25.0 (16.0, 40.0) | 30.0 (19.0, 62.0) | 0.017 |
| Lactate Dehydrogenase, (U/L) | 236.5 (193.0, 275.0) | 227.0 (193.0, 262.0) | 0.466 |
| Monocyte Percentage, n (%) | 5.9 (4.5, 7.4) | 5.6 (4.2, 7.1) | 0.465 |
| Basophil Percentage, n (%) | 0.1 (0.1, 0.2) | 0.1 (0.1, 0.2) | 0.038 |
| Urea, (mmol/L) | 5.5 (4.1, 7.5) | 5.7 (4.2, 8.0) | 0.772 |
| Mean Corpuscular Hemoglobin, (pg) | 29.8 (28.8, 31.0) | 30.1 (29.3, 31.1) | 0.198 |
| Mean Corpuscular Hemoglobin Concentration, (g/L) | 333.0 (323.0, 340.0) | 333.0 (321.0, 340.0) | 0.957 |
| Mean Platelet Volume, (fL) | 10.7 (10.2, 11.7) | 10.8 (9.8, 11.8) | 0.982 |
| Total Cholesterol, (mmol/L) | 4.2 (3.6, 5.0) | 4.1 (3.5, 4.8) | 0.461 |
