# Supplementary material for: Deep Learning for Dynamic Prognostic Prediction in Minimally Invasive Surgery for Intracerebral Hemorrhage: Model Development and Validation Study
Source: JMIR Med Inform. 2026 Jan 7;14:e86327. doi: 10.2196/86327 (PMC12824578; doi:10.2196/86327)
Supplement: Multimedia Appendix 4 [file medinform_v14i1e86327_app4.docx]

Feature labels are as follows：

Operation day：Gender, Cardiac Arrhythmia, Cerebral Arteriosclerosis, Cerebral Vascular Stenosis, GCS, NIHSS, ICH score, Systolic Blood Pressure, Diastolic Blood Pressure, Pulse Rate, Body Temperature, Respiratory Rate, Drainage Volume, Dose of Thrombolytic Agent, Fibrinogen,

International Normalized Ratio (INR), Prothrombin Time, Activated Partial Thromboplastin Time, Thrombin Time, Alanine Aminotransferase, Aspartate Aminotransferase, Total Bilirubin, Direct Bilirubin, Serum Creatinine, Serum Uric Acid, Serum Total Calcium, Serum Potassium, Serum Sodium, Serum Albumin, Lymphocyte Count, White Blood Cell Count, Hemoglobin, D-Dimer, Total Protein, Platelet Count, Neutrophil Count, Gamma-Glutamyl Transferase, Lactate Dehydrogenase, Monocyte Percentage, Basophil Percentage, Urea, Mean Corpuscular Hemoglobin, Mean Corpuscular Hemoglobin Concentration, Mean Platelet Volume, Total Cholesterol, HCO_3_-, Red Blood Cell Count, Ionized Calcium (pH-Corrected)

1D：Gender, Cardiac Arrhythmia, Cerebral Arteriosclerosis, Cerebral Vascular Stenosis, Diastolic Blood Pressure, Pulse Rate, Body Temperature, Respiratory Rate, Fibrinogen, International Normalized Ratio (INR), Prothrombin Time, Activated Partial Thromboplastin Time, Thrombin Time, Alanine Aminotransferase, Aspartate Aminotransferase,' Total Bilirubin, Direct Bilirubin, Serum Creatinine, Serum Uric Acid, Serum Total Calcium, Serum Potassium, Serum Sodium, Serum Albumin, Lymphocyte Count, White Blood Cell Count, Hemoglobin, D-Dimer, Total Protein, Platelet Count, Neutrophil Count, Gamma-Glutamyl Transferase, Lactate Dehydrogenase, Monocyte Percentage, Basophil Percentage, Urea, Mean Corpuscular Hemoglobin, Mean Corpuscular Hemoglobin Concentration, Mean Platelet Volume, Total Cholesterol, HCO3-, Red Blood Cell Count, Ionized Calcium (pH-Corrected), GCS, NIHSS, ICH score, Drainage Volume, Dose of Thrombolytic Agent

3D：Gender, Cardiac Arrhythmia, Cerebral Arteriosclerosis, Cerebral Vascular Stenosis, Systolic Blood Pressure, Diastolic Blood Pressure, Pulse Rate, Body Temperature, Respiratory Rate, Fibrinogen, International Normalized Ratio (INR), Prothrombin Time, Activated Partial Thromboplastin Time, Thrombin Time, Alanine Aminotransferase, Aspartate Aminotransferase, Total Bilirubin, Direct Bilirubin, Serum Creatinine, Serum Uric Acid, Serum Total Calcium, Serum Potassium, Serum Sodium, Serum Albumin, Lymphocyte Count, White Blood Cell Count, Hemoglobin, D-Dimer, Total Protein, Platelet Count, Neutrophil Count, Gamma-Glutamyl Transferase, Mean Corpuscular Hemoglobin, Mean Corpuscular Hemoglobin Concentration, Mean Platelet Volume, Total Cholesterol, HCO3-, Red Blood Cell Count, Ionized Calcium (pH-Corrected), GCS, NIHSS, ICH score, Drainage Volume, Dose of Thrombolytic Agent

7D：Gender, Cardiac Arrhythmia, Cerebral Arteriosclerosis, Cerebral Vascular Stenosis, Dose of Thrombolytic Agent, Systolic Blood Pressure, Diastolic Blood Pressure, Pulse Rate, Body Temperature, Respiratory Rate, Fibrinogen, International Normalized Ratio (INR), Prothrombin Time, Activated Partial Thromboplastin Time, Thrombin Time, Alanine Aminotransferase, Aspartate Aminotransferase, Total Bilirubin, Direct Bilirubin, Serum Creatinine, Serum Uric Acid, Serum Total Calcium, Serum Potassium, Serum Sodium, Serum Albumin, Lymphocyte Count, White Blood Cell Count, Hemoglobin, D-Dimer, Total Protein, Platelet Count, Neutrophil Count, Gamma-Glutamyl Transferase, Monocyte Percentage, Basophil Percentage, Urea, Mean Corpuscular Hemoglobin, Mean Corpuscular Hemoglobin Concentration, Mean Platelet Volume, Total Cholesterol, HCO3-, Red Blood Cell Count, Ionized Calcium (pH-Corrected), GCS, NIHSS, ICH score

14D：Gender, Cardiac Arrhythmia, Cerebral Arteriosclerosis, Cerebral Vascular Stenosis, Systolic Blood Pressure, Diastolic Blood Pressure, Pulse Rate, Body Temperature, Respiratory Rate

the day of drainage tube removal：Catheterization Duration, Hematoma Expansion, Type of Thrombolytic Agent, Intraventricular Hemorrhage, Urinary Tract Infection, Respiratory Failure, gastrointestinal bleeding, Cerebral Amyloid Angiopathy, Time from Symptom Onset to Surgery, Thalamic Hemorrhage, Lobar Hemorrhage, Subarachnoid Hemorrhage,Age, Single/Dual Catheterization, Basal Ganglia Hemorrhage, History of Antiplatelet/Anticoagulant Therapy, History of Smoking, History of Hypertension
